# SEED: the six excesses (*Liu Yin*) evaluation and diagnosis scale

**DOI:** 10.1186/s13020-015-0059-4

**Published:** 2015-10-27

**Authors:** Pei-Jung Chiang, Tsai-Chung Li, Chih-Hung Chang, Li-Li Chen, Jun-Dai Lin, Yi-Chang Su

**Affiliations:** Graduate Institute of Chinese Medicine, School of Chinese Medicine, China Medical University, Taichung, Taiwan; Department of Traditional Chinese Medicine, Taichung Veterans General Hospital, Taichung, Taiwan; Graduate Institute of Biostatistics, China Medical University, Taichung, Taiwan; Feinberg School of Medicine, Northwestern University, Chicago, USA; School of Nursing, China Medical University, Taichung, Taiwan; Department of Nursing, China Medical University Hospital, Taichung, Taiwan; Department of Healthcare Administration, College of Medical and Health Science, Asia University, Taichung, Taiwan

## Abstract

**Background:**

Infections such as common colds, influenza, acute upper respiratory infections, bacterial gastroenteritis, and urinary tract infections are usually diagnosed according to patients’ signs and symptoms. This study aims to develop a scale for the diagnosis of infectious diseases based on the six excesses *(Liu Yin)* etiological theory of Chinese medicine (CM) by the Delphi method.

**Methods:**

A total of 200 CM-guided diagnostic items measuring signs and symptoms for infectious diseases were compiled from CM literature archives from the *Han* to *Ming* dynasties, CM textbooks in both China and Taiwan, and journal articles from the China Knowledge Resource Integrated Database. The items were based on infections and the six excesses *(Liu Yin)* etiological theory, i.e., *Feng Xie* (wind excess), *Han Xie* (coldness excess), *Shu Xie* (summer heat excess), *Shi Xie* (dampness excess), *Zao Xie* (dryness excess), and *Huo Xie* (fire excess). The items were further classified into the six excess syndromes and reviewed via a Delphi process to reach consensus among CM experts.

**Results:**

In total, 178 items with a mean or median rating of 7 or above on a scale of 1–9 from a panel of 32 experts were retained. The numbers of diagnostic items in the categories of *Feng* (wind), *Han* (coldness), *Shu* (summer heat), *Shi* (dampness), *Zao* (dryness), and *Huo* (fire) syndromes were 15, 22, 25, 37, 17, and 62, respectively.

**Conclusions:**

A CM-based six excesses (*Liu Yin*) evaluation and diagnosis (SEED) scale was developed for the evaluation and diagnosis of infectious diseases based only on signs and symptoms.

## Background

Infections such as common colds, influenza, acute upper respiratory infections, bacterial gastroenteritis, and urinary tract infections are usually diagnosed according to patients’ signs and symptoms, while the diagnosis of pandemic infections such as influenza H1N1 [1–4] and H5N1 [5, 6] must be confirmed by expensive laboratory tests [7–9] or real-time RT-PCR assays of multiple specimens [10]. Pathogen testing in the laboratory might be of low sensitivity [11], and low accuracy in some cases [12–14], and above all time-consuming [15].

**Fig. 1:**
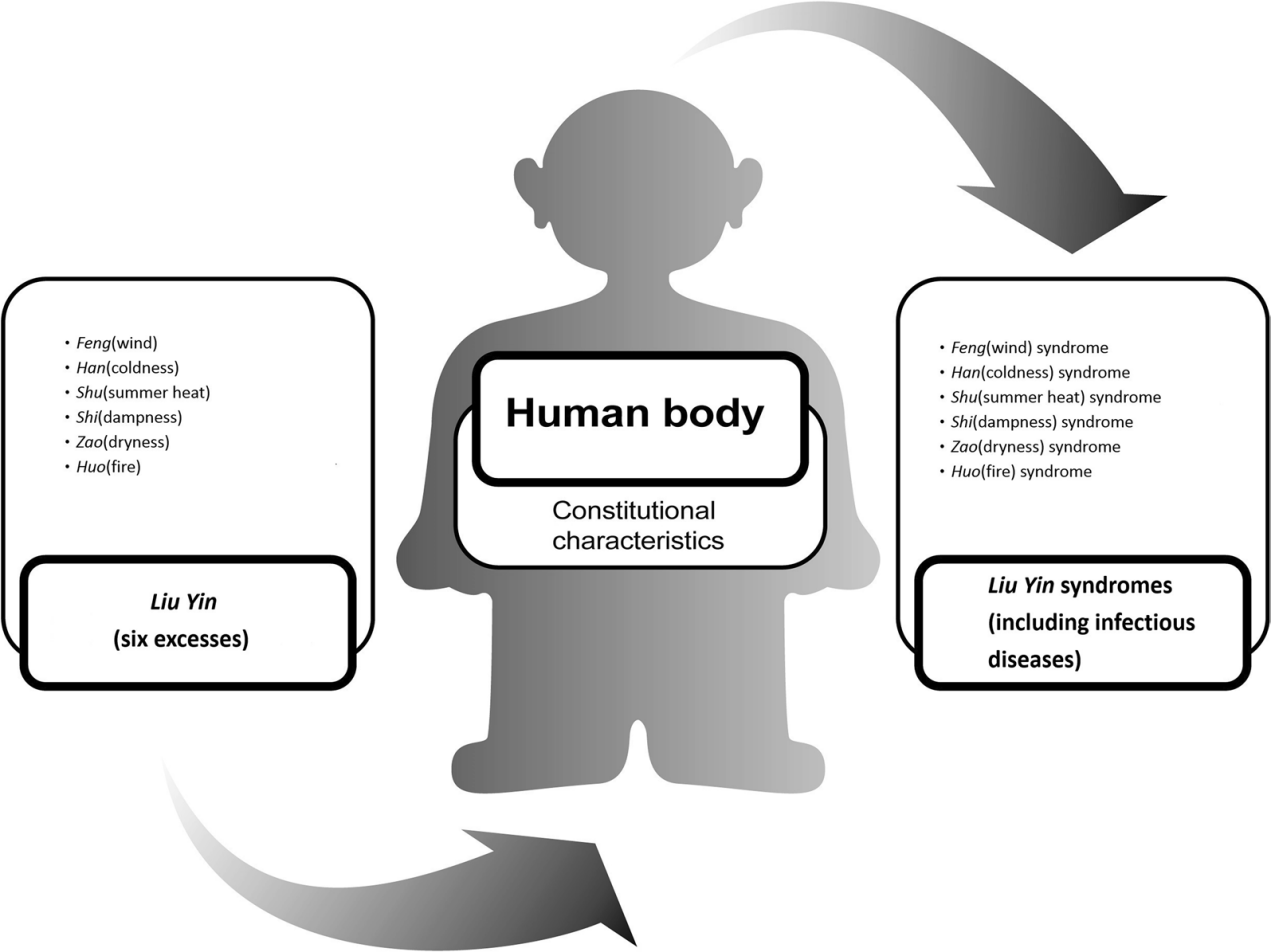
Infectious diseases were classifiable in CM according to the *Liu Yin* (six excesses), *Feng* (wind), *Han* (coldness), *Shu* (summer heat), *Shi* (dampness), *Zao* (dryness), and *Huo* (fire), and could be classified into *Feng* (wind), *Han* (coldness), *Shu* (summer heat), *Shi* (dampness), *Zao* (dryness), and *Huo* (fire) syndromes

Chinese medicine (CM) can detect those infectious diseases mentioned above according to the etiological theory of *Liu Yin* (six excesses), i.e., *Feng Xie* (wind excess) representing varying temperature factors, *Han Xie* (coldness excess) representing falling temperature, *Shu Xie* (summer heat excess) representing rising temperature and humidity, *Shi Xie* (dampness excess) representing rising humidity, *Zao Xie* (dryness excess) representing falling humidity, and *Huo Xie* (fire excess) representing rising temperature [16, 17]. These *Liu Yin* (six excesses) collectively describe the circumstantial influences on *Qi* and *Xue* (blood), encompassing a number of CM diagnostic criteria checked by inquiry, inspection, olfaction, audition, percussion, palpation, and pulse examination (Fig. 1), and facilitate diagnostic and therapeutic decisions [18]. However, there has been no standard diagnostic assessment or measurement scales designed for infectious diseases based on the *Liu Yin* (six excesses) theory [19–28].

This study aims to develop the six excesses (*Liu Yin*) evaluation and diagnosis (SEED) scale for infectious diseases based on the *Liu Yin* (six excesses) etiological theory in CM by a Delphi process among experts. The Delphi method aims to build consensus and generate ideas in research fields [29–41], and is useful for the establishment of diagnostic criteria in clinical medicine. It is a structured communication process to establish the definition of the syndromes, the diagnostic criteria or the staging of the diseases, and the suggested treatments in the medical guidelines [42–48].

## Methods

The Delphi method [49] was used to achieve a group panel consensus on the diagnostic items for the *Liu Yin* (six excesses) syndromes among a panel of experts between 2007 and 2008. A nationally representative panel of Chinese medical experts were invited; only CM experts with good knowledge about CM and modern research methods with master or doctoral degrees, and more than 7 years of practicing experience were invited to join the Delphi panel. Based on this consensus, we carried out further statistical analyses for infectious diseases.

An interdisciplinary advisory board was formed by seven members, including five CM experts, one measurement methodologist, and one statistician. The advisory board selected the participants of the Delphi panel.

Representatives from various education backgrounds, medical disciplines, geographical distributions, and clinical experience were considered for the panel. Finally, a total of 32 CM experts meeting these criteria were invited, and all agreed to participate (Table 1). Of the 32 participants, 20 were from CM departments in medical centers, 6 were from district or regional teaching hospitals, and 6 were from private practices; 7 practiced in Northern region, 11 in central region, 6 in Southern region and 8 in other region. Twenty-one panelists held master degrees, 11 had doctoral degrees in medical sciences, and 26 panelists were teachers in academic institutions. The age (mean ± SD) of the panelists was 43 ± 7.0 years with a median of 42 years. The year of practicing experience (mean ± SD) was 11.3 ± 3.7 years with a median of 10 years.

**Table 1:** Basic characteristics of the 32 CM experts

Age, mean (SD), years		
Average	43 (SD = 7.0)	
Median	42	
Spectrum of practice, mean (SD), years		
Average	11.3 (SD = 3.7)	
Median	10	
Educational background, n (%)		
Master degree	21 (65.6 %)	
Doctorate degree	11 (34.4 %)	
Geographic distribution, n (%)		
Northern	7 (21.9 %)	
Central	11 (34.4 %)	
Southern	6 (18.8 %)	
Other	8 (25.0 %)	
Practice institution, n (%)		
Medical centers	20 (62.5 %)	
District teaching hospitals	5 (15.6 %)	
Regional teaching hospitals	1 (3.1 %)	
Private practices	6 (18.8 %)	
Teaching in an academic institution, n (%)		
Yes	26 (81.3 %)	
No	6 (18.8 %)	

The Delphi process was iterative. We began with a systematic review of traditional CM literature including the Medicine Encyclopedia collected by Kentang Wang (AC1552–1639), 122 published modern textbooks, and 7364 journal articles from the China Knowledge Resource Integrated Database. The search keywords included: *Feng* (wind), *Han* (coldness), *Shu* (summer heat), *Shi* (dampness), *Zao* (dryness), *Huo* (fire), *Wai*-*gan* (external contraction), *Liu Yin* (six excesses), and *Yin* (excess), and we identified and compiled a pool of 200 diagnostic items for the *Liu Yin* (six excesses) syndromes. The CM experts categorized each of these 200 items into one of the *Liu Yin* (six excesses) syndromes, and the results were reviewed and face-validated by the same experts. The numbers of items for each *Yin* (excess) syndrome were: *Feng* (wind), 23; *Han* (coldness), 32; *Shu* (summer heat), 25; *Shi* (dampness), 40; *Zao* (dryness), 18; and *Huo* (fire), 62. We had mailed the items to the panelists and invited them to add any items; the numbers remained the same.

**Fig. 2:**
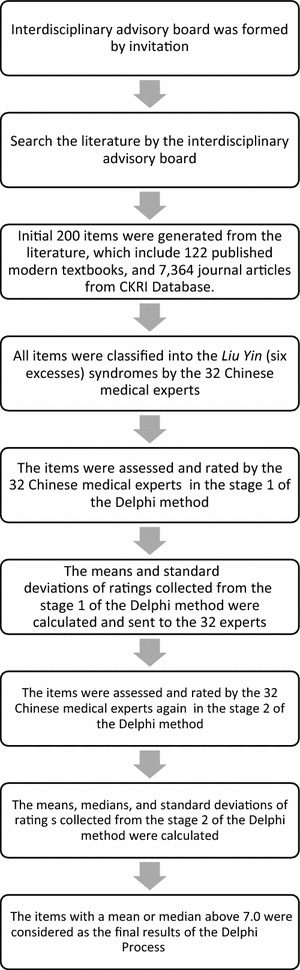
Flow chart of the Delphi process

The classified items were then incorporated into the processes of the Delphi method and circulated via mail to the panelists for their ratings with follow-up phone calls within 2 weeks (Fig. 2). In stage 1, the panelists were asked to rate the appropriateness of each of the classified signs and symptoms on a scale of 1 (highly inappropriate) to 9 (highly appropriate). They were also instructed to provide reasons for the items they rated as “inappropriate”. In stage 2, the results for the mean rating and standard deviation of the individual items were mailed to the panelists, and they were asked again to provide and return their ratings after reviewing the results. The two-stage Delphi method are enough to saturate consensus [49–57], which cannot be maximized by increasing the number of round [58–64]. The progress was assessed by any reduction in the variability of judgments among the panelists. The level of consensus was quantified by the standard deviation of their ratings. After the two-stage Delphi method, the expert-rated diagnostic items with an average or median rating of 7 or above were considered to have face-validity and integrated into the standardized assessment. The cut point of 7.0 had been chosen because it highly represents panelists’ agreement as well as the estimated time required to complete the selected items by CM doctors is expected to be no more than 30 min.

## Results

The mean, median and standard deviation of the ratings for each item from the first and second stages were calculated (Table 2). After the two-stage Delphi method, 15, 22, 25, 37, 17, and 62 diagnostic items with a rating of 7.0 or higher were retained in *Feng* (wind), *Han* (coldness), *Shu* (summer heat), *Shi* (dampness), *Zao* (dryness), and *Huo* (fire) syndromes, respectively. The standard deviations of all 178 retained items decreased from the first stage to the second stage because of the achieved agreement, with the following exceptions: “surging but weak pulse with dipped finger tip” (both 1.02) and “unclear head and eyesight” (1.01–1.06) in *Shu* (summer heat) syndrome; “edematous swelling in face and limbs” (both 1.10) and “unclear head and eyesight” (both 0.67) in *Shi* (dampness) syndrome; and “dry and yellow fur” (1.01–1.06) and “dry, yellow and white fur” (0.61–0.63) in *Huo* (fire) syndrome. However, the upward changes were relatively small.

**Table 2:** Results of the two-stage Delphi method

Syndrome	No.	Item	Stage 1		Stage 2	
			Mean (SD)	Median	Mean (SD)	Median
*Feng* (wind) syndrome	1	Aversion to wind^a^	8.03 (1.06)	8	8.28 (0.96)	8
	2	Floating pulse^a^	7.63 (1.41)	7	7.78 (0.87)	7
	3	Itchy throat^a^	7.25 (1.16)	7	7.41 (0.80)	7
	4	Headache^a^	7.09 (1.30)	6	7.16 (0.51)	6.5
	5	Headache and painful stiffness in nape^a^	7.06 (1.34)	7	7.06 (0.80)	7
	6	Floating and relaxed pulse^a^	7.00 (1.42)	6	7.28 (0.82)	7
	7	Clear snivel^a^	6.97 (1.23)	7	7.09 (0.69)	7
	8	Sneezing^a^	6.94 (1.34)	5	7.13 (0.79)	5
	9	Pain at unfixed location	6.88 (2.03)	6	6.91 (1.09)	6
	10	Painful stiffness in nape and back	6.84 (1.22)	6	6.84 (0.95)	5
	11	Floating and rapid pulse^a^	6.81 (1.42)	7	7.19 (0.82)	7
	12	Generalized pain^a^	6.75 (1.59)	7	7.06 (0.67)	7
	13	Nasal congestion^a^	6.69 (1.31)	7	7.13 (0.75)	7
	14	Cough, productive^a^	6.59 (1.46)	7	7.06 (0.67)	7
	15	Floating and weak pulse^a^	6.53 (1.65)	7	6.59 (1.07)	7
	16	Aches and pains in joints	6.44 (1.39)	5.5	6.69 (0.59)	5
	17	Generalized itching	6.44 (1.68)	5.5	6.44 (1.37)	5
	18	Thin white fur	6.38 (1.79)	6	6.50 (1.05)	6
	19	Pale red tongue	6.28 (1.90)	6	6.41 (0.98)	6
	20	Deviated eye and mouth	6.16 (1.92)	8	5.84 (1.44)	8
	21	Opisthotonos^a^	5.63 (2.23)	7	5.09 (1.40)	7
	22	Trismus (lockjaw)^a^	5.53 (2.17)	7	5.06 (1.37)	7
	23	Hemiplegia	5.22 (2.35)	6	5.13 (1.43)	6
*Han* (coldness) syndrome	1	Aversion to cold^a^	8.22 (0.87)	8.5	8.41 (0.61)	8
	2	Floating and tight pulse^a^	7.66 (1.10)	7	7.78 (0.79)	7
	3	Preference for hot drinks^a^	7.53 (1.37)	7	7.53 (0.67)	7
	4	Clear phlegm^a^	7.53 (0.98)	7	7.47 (0.57)	7
	5	Clear snivel^a^	7.41 (0.95)	6	7.50 (0.67)	7
	6	A lot of clear and white phlegm^a^	7.38 (1.07)	7	7.34 (0.60)	7
	7	Generalized pain^a^	7.13 (1.34)	7	7.41 (0.67)	7
	8	Headache and painful stiffness in nape^a^	7.13 (1.18)	7	7.28 (0.58)	7
	9	Shivering^a^	7.06 (1.37)	7	7.22 (0.91)	7
	10	Ache in limbs^a^	7.00 (1.39)	7	7.28 (0.73)	7
	11	Cold body and limbs^a^	7.00 (1.87)	7	7.28 (0.96)	7
	12	Tight pulse^a^	6.94 (1.39)	6	7.13 (1.16)	6
	13	Aches and pains in joints^a^	6.88 (1.50)	6	7.28 (0.58)	6
	14	White and moist fur	6.88 (1.50)	6	6.81 (1.00)	6
	15	Not thirsty^a^	6.84 (1.32)	6	7.03 (0.93)	6
	16	White, moist and thin fur^a^	6.84 (1.51)	7	6.94 (0.95)	7
	17	No desire to drink	6.84 (1.27)	6	6.81 (0.90)	6
	18	Clear urine^a^	6.81 (1.64)	7	6.78 (0.75)	7
	19	Painful stiffness in nape and back^a^	6.78 (1.72)	7	6.91 (0.95)	7
	20	White and thin fur	6.78 (1.72)	6	6.84 (0.95)	6
	21	Absence of sweating^a^	6.69 (1.64)	7	7.13 (1.01)	7
	22	Cold feeling on nape and back	6.69 (1.45)	7	6.94 (0.76)	6
	23	Headache^a^	6.63 (1.36)	7	7.06 (0.95)	8
	24	Cough, productive	6.53 (1.24)	7	6.75 (0.72)	6
	25	White fur	6.53 (1.78)	7	6.59 (1.01)	6
	26	Sloppy stool	6.50 (1.32)	7	6.66 (1.00)	6
	27	Reversal cold of extremities	6.50 (1.80)	7	6.59 (1.24)	6
	28	White and slippery fur^a^	6.44 (1.63)	7	6.50 (1.02)	7
	29	Nasal congestion	6.38 (1.36)	7	6.81 (0.63)	6
	30	Sneezing	6.38 (1.34)	6	6.81 (0.64)	6
	31	Lumbar pain or ache^a^	6.13 (1.54)	8	6.53 (0.67)	8
	32	Breathlessness^a^	5.88 (1.52)	7	6.22 (1.10)	7
*Shu* (summer heat) syndrome	1	Thirsty^a^	7.97 (1.00)	8	7.88 (0.71)	8
	2	Fever^a^	7.91 (1.09)	8	8.00 (0.80)	8
	3	High fever^a^	7.84 (1.05)	8	7.88 (0.66)	8
	4	Profuse sweating^a^	7.81 (1.57)	8	7.53 (0.92)	7
	5	Surging pulse^a^	7.66 (0.94)	8	7.66 (0.79)	7
	6	Fatigue^a^	7.59 (1.21)	7	7.72 (0.73)	7
	7	Aversion to heat^a^	7.56 (1.41)	7	7.72 (0.73)	7
	8	Reddish yellow urine^a^	7.56 (0.88)	7	7.47 (0.67)	7
	9	Red tongue^a^	7.53 (0.92)	6	7.63 (0.66)	6
	10	Surging but weak pulse with dipped finger tip^a^	7.53 (1.02)	6	7.28 (1.02)	6
	11	Desire to drink^a^	7.47 (1.41)	7	7.53 (0.67)	7
	12	Unclear head and eyesight^a^	7.44 (1.01)	7	7.03 (1.06)	7
	13	Rapid pulse^a^	7.34 (1.29)	8	7.28 (0.68)	8
	14	Heavy or tired limbs^a^	7.31 (1.38)	8	7.25 (0.84)	7
	15	Reddened complexion^a^	7.28 (0.85)	7	7.22 (0.61)	7
	16	Preference for cold drinks^a^	7.28 (1.51)	7	7.13 (0.87)	7
	17	Shortness of urine^a^	7.22 (1.04)	6	7.06 (0.95)	6
	18	Red lips^a^	7.19 (0.74)	7	7.19 (0.64)	6
	19	Agitation^a^	7.13 (1.04)	7	7.00 (0.76)	7
	20	Lethargy^a^	7.03 (1.26)	7	6.78 (0.87)	7
	21	Dry tongue with little saliva^a^	7.00 (0.95)	8	7.09 (0.93)	7
	22	Anorexia^a^	6.81 (1.15)	8	6.81 (0.90)	8
	23	Dizziness^a^	6.50 (1.22)	8	6.47 (1.08)	8
	24	Stomach reflux^a^	6.38 (1.07)	7	6.34 (0.90)	7
	25	Headache^a^	6.19 (1.51)	7	6.38 (1.10)	7
*Shi* (dampness) syndrome	1	Heavy or tired limbs^a^	8.19 (0.86)	7	8.09 (0.73)	7
	2	Heavy-headedness^a^	8.09 (0.86)	8	8.06 (0.76)	8
	3	Fatigue^a^	7.81 (0.90)	7	7.81 (0.74)	7
	4	White and slimy fur^a^	7.75 (1.05)	7	7.72 (0.68)	7
	5	Unclear head and eyesight^a^	7.50 (0.67)	8	7.50 (0.67)	8
	6	Viscous feeling in oral cavity^a^	7.50 (0.95)	7	7.47 (0.62)	7
	7	Thick and slimy fur^a^	7.47 (1.16)	7	7.63 (0.79)	7
	8	Soggy pulse^a^	7.41 (1.34)	8	7.47 (1.02)	8
	9	Edematous swelling in face and limbs^a^	7.41 (1.10)	7	7.13 (1.10)	7
	10	Edema, generalized	7.28 (1.53)	6	6.94 (0.95)	6
	11	Rash	7.25 (1.30)	6	6.97 (1.09)	6
	12	Sloppy stool^a^	7.22 (1.21)	7	7.38 (0.61)	7
	13	Slippery pulse^a^	7.19 (1.06)	7	7.22 (0.79)	7
	14	Soggy and relaxed pulse^a^	7.19 (1.35)	7	7.19 (0.90)	7
	15	White and slippery fur^a^	7.19 (1.40)	6	7.19 (0.74)	6.5
	16	No desire to drink^a^	7.19 (1.23)	6	7.00 (0.88)	7
	17	Yellow and slimy fur^a^	7.19 (1.18)	7	6.97 (1.03)	7
	18	Slippery fur^a^	7.13 (1.43)	7	7.16 (0.72)	7
	19	Soft stool^a^	6.97 (1.36)	6	7.22 (0.61)	7
	20	Anorexia^a^	6.97 (1.40)	6	7.22 (0.71)	6
	21	Dizziness^a^	6.94 (1.46)	7	7.13 (0.83)	7
	22	Lethargy^a^	6.94 (1.34)	7	7.09 (0.89)	7
	23	White and moist fur^a^	6.94 (1.32)	7	6.97 (0.78)	7
	24	Stool with discharge^a^	6.94 (1.32)	7	6.94 (0.72)	7
	25	Oppression in chest^a^	6.91 (1.42)	7	7.13 (0.83)	7
	26	White and moist fur^a^	6.91 (1.53)	7	7.13 (0.75)	7
	27	Not thirsty^a^	6.91 (1.17)	7	6.97 (0.78)	7
	28	Soggy and rapid pulse^a^	6.91 (1.23)	7	6.97 (0.90)	7
	29	Dyspepsia	6.91 (1.42)	7	6.81 (1.00)	6
	30	Jaundice^a^	6.91 (1.47)	7	6.78 (1.16)	7
	31	Mild fever^a^	6.88 (1.31)	7	7.09 (0.59)	7
	32	Stomach reflux^a^	6.78 (1.24)	8	6.84 (0.81)	8
	33	Gastric stuffiness^a^	6.75 (1.46)	7	7.06 (0.76)	7
	34	Vaginal discharge^a^	6.75 (1.46)	8	7.06(0.76)	8
	35	A lot of clear and white phlegm^a^	6.75 (1.34)	7	7.00 (0.51)	7
	36	Aches and pains in joints^a^	6.72 (1.35)	7	7.03 (0.47)	7
	37	White fur^a^	6.66 (1.49)	7	6.91 (0.78)	7
	38	Vomiting^a^	6.59 (1.24)	7	6.56 (0.98)	7
	39	Ache in limbs^a^	6.50 (1.32)	7	6.72 (0.58)	7
	40	Generalized pain^a^	6.44 (1.48)	6	6.75 (0.50)	7
*Zao* (dryness) syndrome	1	Dry throat^a^	8.16 (0.72)	8	8.17 (0.66)	8
	2	Dry nasal cavity^a^	8.06 (0.84)	7	7.90 (0.49)	6
	3	Dry lips^a^	8.06 (0.88)	8	7.79 (0.49)	8
	4	Dry skin^a^	7.84 (0.92)	8	7.83 (0.54)	8
	5	Dry tongue with little fluid^a^	7.75 (1.05)	8	7.66 (0.55)	8
	6	Cough, nonproductive^a^	756 (1.08)	6	7.62 (0.62)	6
	7	Thirsty^a^	7.38 (1.43)	8	7.24 (0.83)	7.5
	8	Hard bound or dry stool^a^	7.31 (1.28)	7	7.14 (0.49)	7
	9	Sticky phlegm^a^	7.31 (1.03)	7	7.14 (0.52)	7
	10	Dry, thin and white fur^a^	7.25 (1.34)	7	7.17 (0.80)	7
	11	Dry, yellow and white fur^a^	7.25 (1.16)	8	7.00 (0.89)	8
	12	Dry and yellow fur^a^	7.06 (1.29)	7	6.79 (0.73)	7
	13	Desire to drink^a^	7.00 (1.37)	7	7.10 (0.86)	7
	14	Red tongue	6.97 (1.58)	6	6.90 (0.62)	6
	15	Hoarseness^a^	6.88 (1.21)	7	7.00 (0.53)	7
	16	Pruritus^a^	6.81 (1.38)	7	7.10 (0.41)	7
	17	Dyschezia^a^	6.50 (1.34)	7	6.59 (0.95)	7
	18	Cough, productive^a^	6.28 (1.17)	7	6.59 (0.82)	7
*Huo* (fire) syndrome	1	High fever^a^	7.97 (0.82)	8	8.00 (0.57)	8
	2	Fever^a^	7.94 (1.08)	8	7.97 (0.54)	8
	3	Rapid pulse^a^	7.94 (1.19)	8	7.84 (0.68)	8
	4	Heat intolerance^a^	7.91 (0.86)	7	7.94 (0.72)	7
	5	Thirsty^a^	7.91 (1.06)	7	7.78 (0.61)	7
	6	Red tongue^a^	7.88 (1.01)	7	7.75 (0.67)	7
	7	Preference for cold drinks^a^	7.69 (1.00)	6	7.63 (0.66)	6
	8	Surging pulse^a^	7.66 (1.18)	7	7.56 (0.72)	7
	9	Reddish yellow urine^a^	7.63 (1.01)	7	7.59 (0.56)	7
	10	Sore throat^a^	7.56 (1.11)	5	7.69 (0.69)	5
	11	Reddish eye^a^	7.56 (1.01)	7	7.56 (0.80)	7
	12	Desire to drink^a^	7.56 (1.11)	7	7.53 (0.57)	7
	13	Reddened complexion^a^	7.56 (0.84)	7	7.50 (0.72)	6
	14	Erythema, blister or ulcer^a^	7.53 (0.72)	7	7.47 (0.98)	7
	15	Hard bound or dry stool^a^	7.50 (1.19)	7	7.44 (0.67)	7
	16	Agitation^a^	7.50 (1.11)	6	7.38 (0.71)	6
	17	Red lips^a^	7.47 (1.02)	6	7.63 (0.71)	6
	18	Sore swollen gum^a^	7.47 (1.02)	7	7.44 (0.76)	6
	19	Dry and yellow fur^a^	7.44 (0.56)	7	7.47 (0.62)	8
	20	Hotness in chest^a^	7.44 (1.01)	7	7.38 (0.55)	7
	21	Red tip of tongue^a^	7.44 (1.05)	7	7.38 (0.71)	7
	22	Red tip and margin of tongue^a^	7.44 (1.05)	7	7.38 (0.71)	7
	23	Red dot on tongue^a^	7.41 (1.07)	8	7.41 (0.67)	8
	24	Floating and rapid pulse^a^	7.38 (1.18)	7	7.41 (0.67)	7
	25	Dry tongue with little fluid^a^	7.38 (1.01)	7	7.31 (0.64)	7
	26	Dry, yellow and white fur^a^	7.38 (0.61)	7	7.28 (0.63)	7
*Huo* (fire) syndrome	27	Ulcer on tongue^a^	7.34 (1.26)	6	7.31 (0.74)	6
	28	Aphtha^a^	7.34 (1.15)	8	7.28 (0.68)	8
	29	Hot feeling around anus^a^	7.31 (1.09)	7	7.28 (1.02)	7
	30	Dry lips^a^	7.31 (1.38)	7	7.16 (0.57)	7
	31	Profuse sweating^a^	7.31 (1.00)	8	7.06 (0.88)	8
	32	Purpura^a^	7.31 (1.28)	7	7.06 (0.76)	7
	33	String-like and rapid pulse^a^	7.28 (1.14)	8	7.16 (0.63)	7
	34	Stinky diarrhea^a^	7.28 (1.16)	7	6.97 (0.61)	7
	35	Slippery and rapid pulse^a^	7.22 (1.16)	7	7.22 (0.61)	7
	36	Hotness in abdomen^a^	7.22 (1.21)	7	7.19 (0.54)	7
	37	Dry throat^a^	7.22 (1.29)	7	7.16 (0.57)	6
	38	Decreased urination^a^	7.22 (1.04)	6	7.09 (0.69)	6
	39	Dysuria^a^	7.22 (0.97)	7	6.91 (0.93)	7
	40	Skin wheal^a^	7.16 (0.92)	8	7.16 (0.72)	8
	41	Yellow phlegm^a^	7.13 (1.24)	7	7.22 (0.55)	7
	42	Epistaxis^a^	7.13 (1.36)	7	7.22 (0.66)	7
	43	Sudden and watery diarrhea^a^	7.13 (1.01)	7	6.97 (1.12)	7
	44	Yellow snivel^a^	7.06 (1.37)	8	7.16 (0.63)	7
	45	Dyschezia^a^	7.06 (1.22)	6.5	7.09 (0.64)	6
	46	Anguish in heart^a^	7.03 (1.26)	7.5	7.19 (0.64)	7
	47	Dry nasal cavity^a^	7.00 (1.30)	7	7.09 (0.53)	7
	48	Bitter taste in mouth^a^	7.00 (1.34)	7	7.06 (0.80)	7
	49	Rash^a^	6.97 (1.40)	7	7.03 (0.69)	7
	50	Bloody stool with pus^a^	6.97 (0.93)	7	6.97 (1.09)	7
	51	Difficulty falling asleep^a^	6.94 (1.32)	7	7.09 (0.69)	7
	52	Delirious speech^a^	6.84 (1.42)	8	6.72 (0.99)	8
	53	Gingival bleeding^a^	6.81 (1.40)	7	6.91 (1.03)	7
	54	Nasal flaring^a^	6.75 (1.46)	7	6.81 (1.06)	7
	55	Difficulty maintaining sleep^a^	6.72 (1.30)	7	6.91 (0.59)	7
	56	Hemoptysis^a^	6.69 (1.40)	7	6.84 (0.85)	7
	57	Hematuria^a^	6.69 (1.49)	7	6.66 (1.10)	7
	58	Headache^a^	6.56 (1.32)	8	6.41 (0.87)	7
	59	Hematemesis^a^	6.44 (1.26)	8	6.44 (0.94)	8
	60	Breathless^a^	6.34 (1.26)	7.5	6.13 (0.94)	7
	61	Lethargy^a^	6.31 (1.60)	7	6.31 (1.12)	7
	62	Tinnitus^a^	5.84 (1.65)	7	5.94 (0.91)	7

The translations were mainly according to “*WHO International Standard Terminologies on Traditional Medicine in the Western Pacific Region* [70]”

*SD* standard deviation

^a^The 178 diagnostic items graded as 7.0 and above

The total number of retained items in each of the *Liu Yin* (six excesses) syndromes varied from 15 items in *Feng* (wind) syndrome to 62 items in *Huo* (fire) syndrome. All syndromes comprised two kinds of diagnostic items: subjective discomfort factors and objective examinations by the patients themselves or by clinicians. The diagnostic items for subjective discomfort included the following: items regarding sleeping, appetite, eyesight, and hearing; items regarding behavioral adjustments such as aversion, preference, anguish, and agitation; items regarding sensations of coldness, hotness, dryness, and bitterness; and items regarding feelings of pain, itch, ache, congestion, thirst, viscousness, oppression, fullness, stuffiness, heaviness, and tiredness. The diagnostic items for objective examinations included the following: general symptoms regarding complexion, eyes, lips, tongue, skin, snivel, stool, urine, phlegm, and awareness; physical responses such as breathlessness, coughing, shivering, sweating, sneezing, and vomiting; and appearance changes such as hemiplegia, deviated eyes and mouth, opisthotonos, trismus, and edema. In addition to the two kinds of diagnostic items, 4, 2, 3, 4, and 5 items of pulse examination were retained in *Feng* (wind), *Han* (coldness), *Shu* (summer heat), *Shi* (dampness), and *Huo* (fire) syndromes, respectively.

Some items were retained in more than one syndrome because of their overlapping contributions, as follows: “headache”, “generalized pain”, “clear snivel”, and “headache and painful stiffness in nape” in *Feng* (wind) and *Han* (coldness) syndromes; “floating and rapid pulse” in *Feng* (wind) and *Huo* (fire) syndromes; “aches and pains in joints” and “a lot of clear and white phlegm” in *Han* (coldness) and *Shi* (dampness) syndromes; “fatigue”, “heavy or tired limbs”, and “unclear head and eyesight” in *Shu* (summer heat) and *Shi* (dampness) syndromes; “thirsty”, “desire to drink”, and “dry tongue with little saliva” in *Shu* (summer heat), *Zao* (dryness), and *Huo* (fire) syndromes; “dry nasal cavity”, “dry lips”, “dry throat”, “hard bound or dry stool”, and “dry, yellow and white fur” in *Zao* (dryness) and *Huo* (fire) syndromes; and “aversion to heat”, “fever”, “high fever”, “profuse sweating”, “red lips”, “preference for cold drinks”, “agitation”, “shortness of urine”, “reddish yellow urine”, “red tongue”, “surging pulse”, and “rapid pulse” in *Shu* (summer heat) and *Huo* (fire) syndromes. Including these overlapping items, the SEED scale for infectious diseases, which captured the *Liu Yin* (six excesses) syndromes, was created and formatted as follows: *Feng* (wind), 15 items; *Han* (coldness), 22 items; *Shu* (summer heat), 25 items; *Shi* (dampness), 37 items; *Zao* (dryness), 17 items; and *Huo* (fire), 62 items. Following the integration of common items, a total of 102 diagnostic criteria conformed to the *Liu Yin* (six excesses) and covered the manifestations of the *Liu Yin* (six excesses) syndromes.

## Discussion

This study codified the *Liu Yin* (six excesses) syndromes by the Delphi method with a panel of 32 CM experts. The 178 diagnostic items derived from the two-stage Delphi method combined information gleaned from CM classic textbooks, journal articles, and opinions of CM experts. All six syndromes encompassed diagnostic criteria regarding subjective discomfort and objective examinations, both were deemed essential for diagnostic judgment. Subjective discomfort was important for medical care considerations, while objective examinations were crucial for disease progress evaluations.

Common diagnostic items were present in different syndromes as a result of the same body responses to different *Yin* (excess). For example, “generalized pain” in *Feng* (wind) and *Shi* (dampness) syndromes results from obstructed circulation of *Qi* and *Xue* (blood) [65]. Common items combined with different signs or symptoms imply different body responses [66, 67]. “Fatigue” combined with “thirsty” was attributed to *Shu Xie* (summer heat excess), while “fatigue” combined with “not thirsty” was attributed to *Shi Xie* (dampness excess).

Five syndromes, except for *Feng* (wind) syndrome, comprised diagnostic items of tongue examinations for detecting the *Qi* and *Xue* (blood) status. Five syndromes, except for *Zao* (dryness) syndrome, comprised pulse examinations for instant diagnostic judgment in CM practice [68]. Clinical skills in pulse and tongue examinations were important for accurate assessment of signs and symptoms in the SEED scale [69], just as in CM clinical practice.

The 102 diagnostic criteria were checked by inquiry, inspection, olfaction, audition, percussion, palpation, and pulse examination to ensure complete consideration in medical care. Arrangement of these diagnostic items in a more systematic manner along with a designed record format for tongue and pulse examinations would be necessary for clinical practice. Practice manuals proposing the definition, description, and standardized process for each diagnostic criterion have also been developed for correct implementation.

The limitation of homogeneous education backgrounds is inevitable, since we excluded CM clinicians who had only practicing experience, but no postgraduate degrees from participating in the Delphi panel, as some research background would be required for understanding of the Delphi method and the statistical results circulated during the processes. The panelists’ average practicing experience of 11.7 years was considered to be a good representation of their clinical experience.

Because the *Liu Yin* (six excesses) syndromes were general categories, exploratory and confirmatory factor analyses were conducted to derive and validate the underlying structures of the SEED scale and to reveal the correlations among signs and symptoms. Future studies, including clinical observations to avoid item redundancy and to confirm the clinical practice of the SEED scale in infectious diseases, are required.

It’s the first scale based on the six excesses (*Liu Yin*) and constructed by the Delphi method; the CM experts epitomized the contents of CM literature and journal articles via the process. Future applications of the SEED scale in clinical practice, research and CM education are expected.

## Conclusion

A CM-based SEED scale was developed for the evaluation and diagnosis of infectious diseases based on only signs and symptoms.
